# Supplementary material for: A stochastic compartmental model to simulate intra- and inter-species influenza transmission in an indoor swine farm
Source: PLoS One. 2023 May 4;18(5):e0278495. doi: 10.1371/journal.pone.0278495 (PMC10159208; doi:10.1371/journal.pone.0278495)
Supplement: S1 Table — *to calculate days to first Workforce infection and the deterministic nature of these models we only used iterations in which to total infected workforce count exceeded 0.5. (PDF) [file pone.0278495.s001.pdf]

Supplemental Table 1 Epistemic Uncertainty Analysis Results

		Model outputs				Realized Model Parameter Values (Sensitivity Analysis)		
Control Measure (CM)	CM variable value	Total Pigs Infected [95% Variable interval] (Deterministic)	Median Total Pigs infected (Stochastic)	Days to first Workforce Infection [95% Variable interval] (Deterministic)*	Median days to first Workforce infection (Stochastic)	Median Beta Hog [95% Variable interval]	Median Beta H2W [95% Variable interval]	Median Beta W2H [95% Variable interval]
Baseline Model	No Control Measures	3966.91 [3950.13 - 3972.07	3957	10.8 [6.1 – 18.35]	20.9	0.007 [0.001 - 0.016]	5.05e-05 [5.89e-06 - 9.48e-05]	5.13e-05 [6.16e-06 - 9.55e-05
Mass Vaccination	20%	3173.02 [3148.23 - 3177.38	3161	11.6 [6.9 – 20]	23.6	0.007 [0.001 - 0.016]	5.00e-05 [5.73e-06 - 9.55e-05]	5.05e-05 [5.93e-06 - 9.49e-05]
	40%	2378.72 [2325.83 – 2382.53]	2362	13.6 [7.7 – 22.91]	28.4	0.007 [0.001 - 0.016]	5.06e-05 [6.08e-06 - 9.499e-05]	5.07e-05 [6.58e-06 - 9.54e-05]
	60%	1584.97 [1465.33 – 1588]	1540	15.4 [9.1 – 24.95]	38.0	0.007 [0.002 - 0.02]	5.05e-05 [5.77e-06 - 9.49e-05]	5.08e-05 [6.04e-06 - 9.52e-05]
Isolation	0.33	3965.94 [3646.02 - 3971.74]	3950	11.7 [6.7 – 21.2]	24.0	0.007 [0.001 - 0.016]	5.08e-05 [5.48e-06 - 9.491e-05]	5.14e-05 [5.96e-06 - 9.52e-05]
	0.5	3964.49 [3297.26 - 3971.63]	3945	12.9 [7.2 – 23.8]	25.6	0.007 [0.002 - 0.02]	4.94e-05 [6.42e-06 - 9.52e-05]	5.08e-05 [6.36e-06 - 9.44e-05]
	1.0	3948.46 [1436.2 - 3970.62]	2	15.2 [8.2 – 27.69]	29.9	0.007 [0.002 - 0.02]	5.16e-05 [6.69e-06 - 9.54e-05]	5.03e-05 [6.01e-06 - 9.46e-05]
Workforce Flow	Room 4	3946.89 [790.9 - 3970.72	3956	15.1 [7.9 – 29.85]	20.8	0.007 [0.001 - 0.02]	4.93e-05 [5.25e-06 - 9.49e-05]	4.97e-05 [6.32e-06 - 9.516e-05]

	Room 3	3958.86 [1214.25 – 3982]	2974	15.45 [8.65 – 28.8]	21.0	0.007 [0.002 - 0.02]	5.03e-05 [6.15e-06 - 9.47e-05]	5.05e-05 [6.01e-06 - 9.48e-05]
	Room 2	3963.59 [971.97 - 3989.08]	1987	16.45 [8.91 – 30.74]	19.8	0.007 [0.002 - 0.02]	5.11e-05 [6.11e-06 - 9.51e-05]	5.16e-05 [6.33e-06 - 9.51e-05]
	Room 1	3965.7 [1123.92 - 3992.36]	996	17.6 [9.96 – 29.24]	17.4	0.007 [0.002 - 0.02]	5.09e-05 [6.11e-06 - 9.44e-05]	4.92e-05 [5.96e-06 - 9.52e-05]

*to calculate days to first Workforce infection and the deterministic nature of these models we only used iterations in which the total infected workforce count exceeded 0.5
